# An ER-associated miRNA signature predicts prognosis in ER-positive breast cancer

**DOI:** 10.1186/s13046-014-0094-5

**Published:** 2014-11-06

**Authors:** Xin Zhou, Xiaping Wang, Zebo Huang, Lei Xu, Wei Zhu, Ping Liu

**Affiliations:** Department of Oncology, First Affiliated Hospital of Nanjing Medical University, 300 Guangzhou Road, Nanjing, 210029 China; Cancer Center of Nanjing Medical University, Nanjing, 210029 China; Key Laboratory of Human Functional Genomics of Jiangsu Province, Clinical Diabetes Centre of Jiangsu Province, Nanjing Medical University, Nanjing, 210029 China; Department of Thoracic Surgery, The Affiliated Jiangning Hospital of Nanjing Medical University, Nanjing, 210029 China

**Keywords:** Breast cancer, ER status, miRNA signature, prognosis

## Abstract

**Background:**

Breast cancer patients with positive estrogen receptor (ER) have a better prognosis. However, no prognostic miRNA signature was reported in the ER-positive breast cancer. The aim of the study was to identify and assess the prognostic significance of a miRNA signature in ER-positive breast cancer.

**Methods:**

Two cohorts from The Cancer Genome Atlas (TCGA) dataset were used as training (n =596) and testing set (n =319). Differential expression profiling was identified in the training set. And the prognostic value of the miRNA signature was then assessed in the two cohorts.

**Results:**

A total of 14 miRNAs were observed to be associated with the status of ER by significance analysis of microarrays (SAM) in the training set. Patients were characterized as high score or low score group according to the calculated risk scores from each miRNA. And patients in high score group had worse overall survival compared with those in low score group both in the training and testing set.

**Conclusions:**

Our study revealed a miRNA signature including 14 miRNAs associated with ER status which could act as a prognostic marker in ER-positive breast cancer.

**Electronic supplementary material:**

The online version of this article (doi:10.1186/s13046-014-0094-5) contains supplementary material, which is available to authorized users.

## Introduction

Breast cancer is a heterogeneous disease that comprises a range of subgroups with diverse clinical behaviors and responses to treatment [1]. Many breast-cancer-related genes have been investigated to explore the molecular mechanism of carcinogenesis and diverse clinical outcome of the disease [2-6]. Among them, some specific genes such as estrogen receptor (ER) [7], progesterone receptor (PR) [8] and human epidermal growth factor receptor 2 (HER2) [9] have been used to identify different subgroups and indicate different prognostic results with different treatment modalities in the clinical. Patients with ER-positive status which account for almost 70% of breast cancer always had a better prognosis compared with those ER-negative types [10]. However, ER-positive patients also have distinct outcomes and almost 20% might relapse within 10 years after surgery [11]. Thus, there is an urgent need to identify biomarkers that could predict prognostic outcome in patients with ER-positive breast cancer.

MicroRNAs (miRNAs) are short (approximately 22 nucleotides), single-stranded and highly conserved non-coding RNAs which could regulate almost one-third human genome based on either mRNA degradation or translational repression through base pairing with the 3′-untranslated region of target mRNAs at post-transcriptional level [12,13]. Reportedly, miRNAs play important roles in various biological processes, such as cellular development, differentiation, proliferation, angiogenesis and metabolism [14-17]. The prognostic value of miRNAs has been explored in several cancer types, such as colon cancer [18], nasopharyngeal carcinoma [19], hepatocellular carcinoma [20] and glioma [21]. To date, no prognostic miRNA signature for ER-positive breast cancer has been reported. In the present study, we used data retrieved from The Cancer Genome Atlas (TCGA, http://cancergenome.nih.gov/) and identify a miRNA signature associated with the status of ER which could act as a prognostic predicator for ER-positive patients.

## Methods and materials

### Expression profiles

The miRNA expression microarray data (Level 3) and corresponding clinical data for breast cancer patients were obtained from The Cancer Genome Atlas (TCGA) database (http://cancergenome.nih.gov) and Ref [22,23]. The data from two independent platforms were classified into two cohorts. The cohort with 596 patients (456 ER-positive and 140 ER-negative) undergone IlluminaHiSeq_miRNASeq platform and the smaller dataset with 319 cases (251 ER-positive and 68 ER-negative) from IlluminaGA_miRNASeq platform were used as training and validation set, respectively. As the data were obtained from TCGA, further approval by an ethics committee was not required.

### Statistical analysis

The differential expression profile between ER-positive and ER-negative cases in training set was assessed by using significance analysis of microarrays (SAM) on BRB array tools package which was developed by Richard Simon and the BRB-ArrayTools Development Team [24]. And P value <0.001 with fold change (FC) > 2.8 (log2 FC >1.5) was considered significant. Risk score analysis was performed to evaluate the association of ER associated miRNA signature and overall survival of ER-positive patients. ROC curves were used to identify the optimal cutoff value for each miRNA to discriminate ER-positive from negative cases. The score for each miRNA, denoted as S, was set as 1 if the expression level was greater than the cutoff value, otherwise was set as 0 [25]. A risk score formula for predicting survival was developed based on a linear combination of the expression level multiplied regression coefficient derived from the univariate logistic regression model (B) fitted with the status of ER for each significant miRNA: Risk score=${\displaystyle {\sum}_{j-1}^k{S}_{ij}*{B}_j}$. In the equation above, S_*ij*_ is the risk score for miRNA *j* on patient *i*, and B*j* is the weight of the risk score of miRNA *j*. Patients in the training and test set were divided into high score and low score group according to the risk score. Overall survival curves for the two groups were estimated by the Kaplan-Meier methodology and compared using log-rank test.

Survival analyses were performed using SPSS version 16.0 for Windows (Statistical Package for Social Sciences, Chicago, IL). All p values were two-sided and statistical significance was defined as p < 0.05.

## Results

### Identification of ER associated miRNA signature

**Figure 1:**
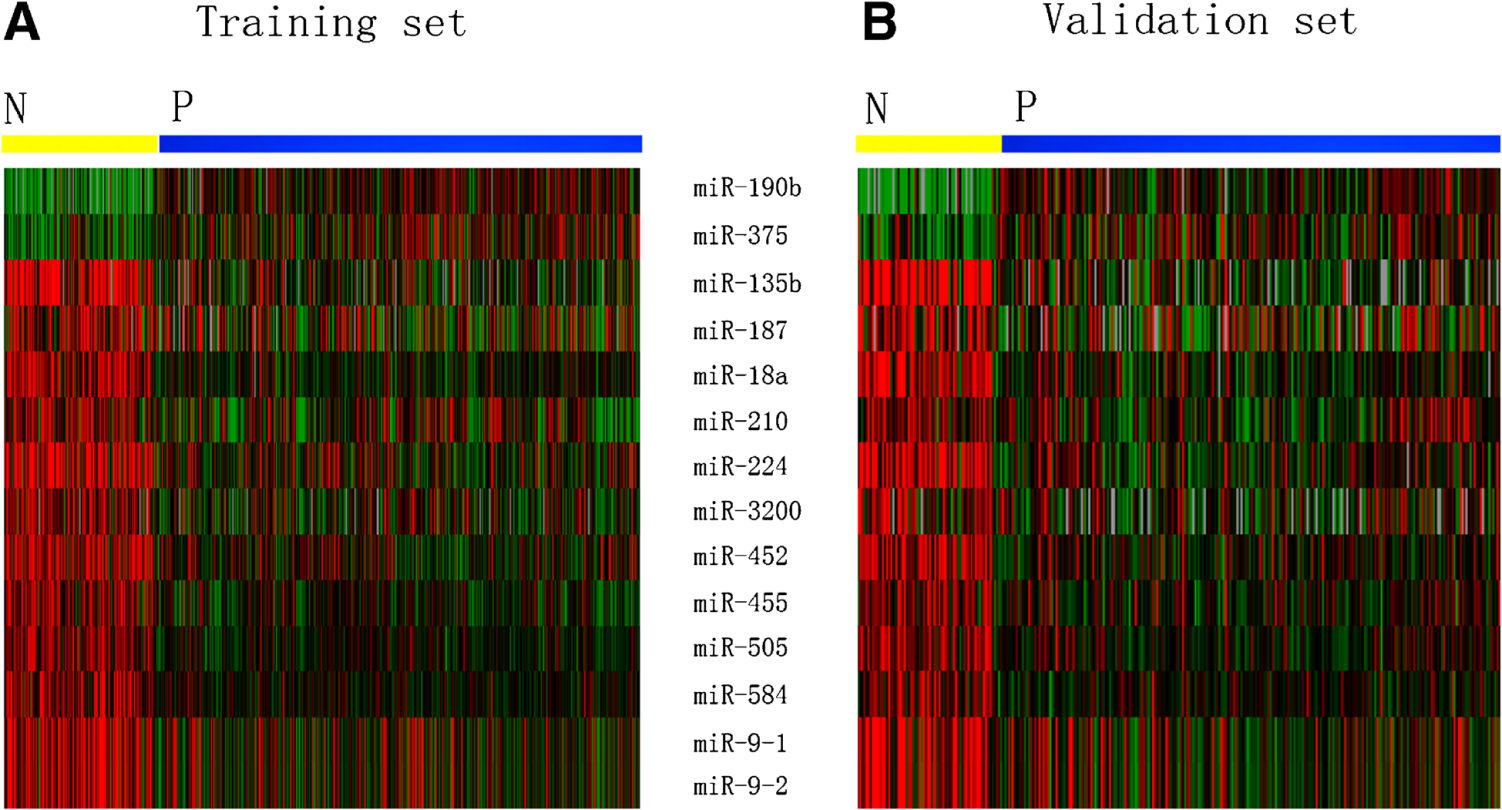
**Different expression of 14 miRNAs associated with ER status in both training set (A) and validation set (B).** N: negative ER; P: positive ER.

A total of 14 miRNAs were identified to be associated with ER status in the training set. Among them, 12 miRNAs (miR-135b, miR-187, miR-18a, miR-210, miR-224, miR-3200, miR-452, miR-455, miR-505, miR-584, miR-9-1 and miR-9-2) were significantly up-regulated while two down-regulated (miR-190b, miR-375) in ER-negative cases compared with ER-positive patients (Figure 1A). As shown in Figure 1B, each of the 14 miRNAs was significantly dysregulated and showed the consistent tendency according to the status of ER in the validation set.

### Prognostic value of ER associated miRNA signature in ER-positive patients

**Figure 2:**
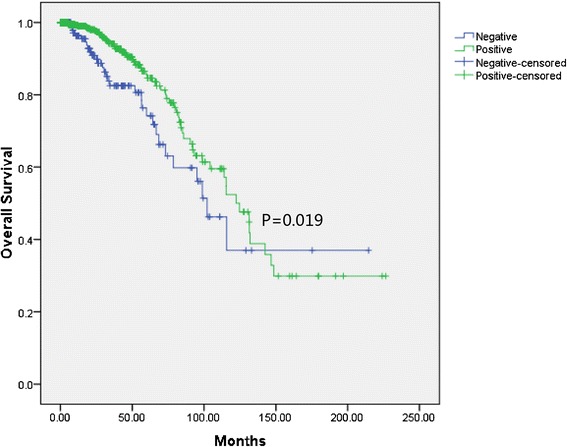
**Overall survival of breast cancer in the combined cohorts according to the status of ER.**

**Figure 3:**
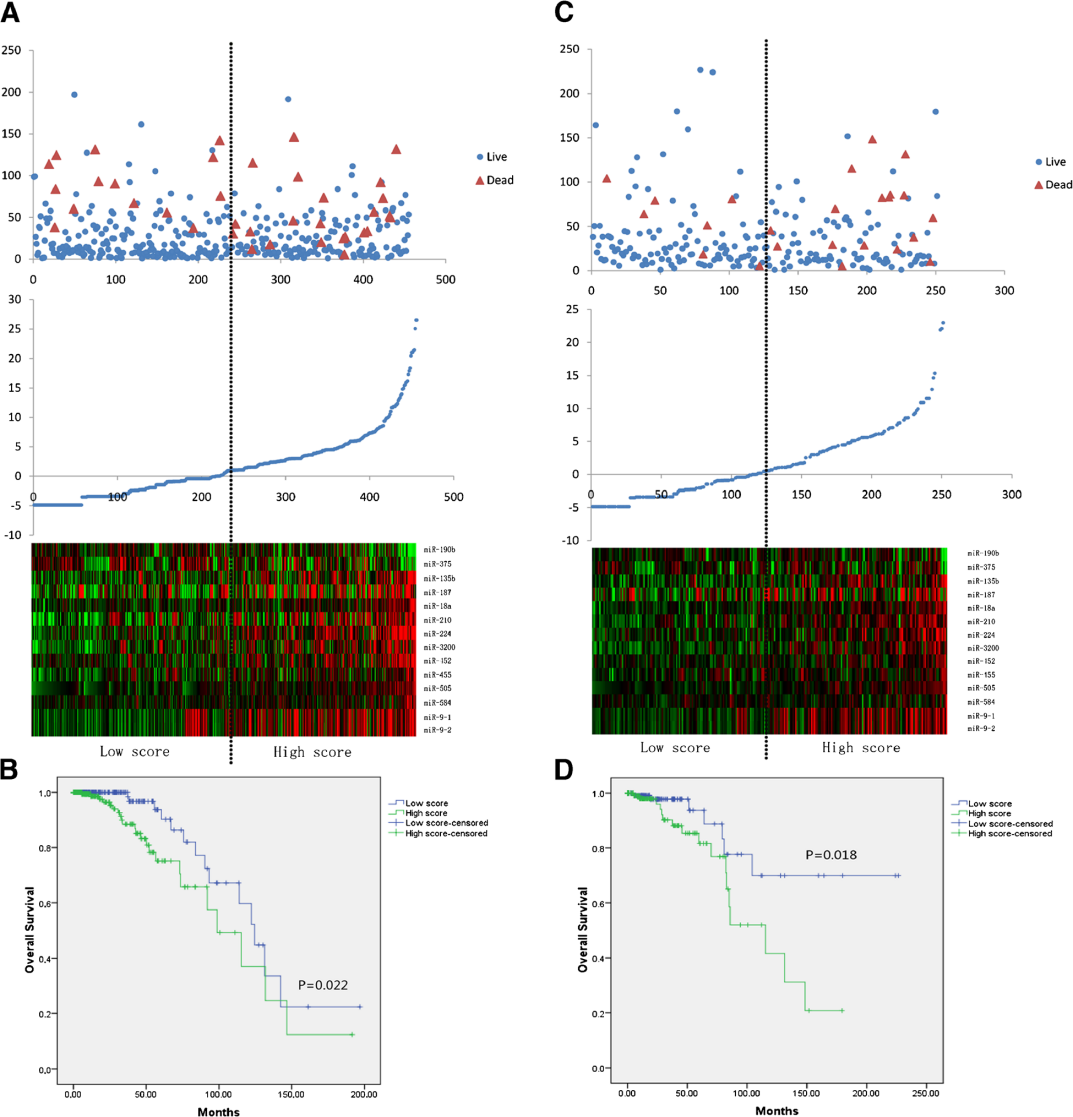
**Risk scores for the ER-associated miRNA signature and outcome in breast cancer patients with positive ER status. (A)** Training set and **(C)** validation set: (Top) survival status and duration of cases; (Middle) risk scores of miRNA signature; (Bottom) low and high score group for the 14 miRNAs; Kaplan-Meier curves for the low score and high score groups in both training set **(B)** and validation set **(D)**. Patients with high score had worse overall survival than those with low score.

By combining cases from the two cohorts, better overall survival could be found (Figure 2) in the ER-positive patients compared with ER-negative cases (P = 0.019). To assess the prognostic value of ER associated miRNA signature, 456 ER-positive cases in training set was divided into high and low score group according to the median risk score (ROC curves for each miRNA were present in the Additional file 1: Figure S1). As shown in Figure 3A, the up-regulated miRNAs identified in ER-negative cases exhibit high expression in high score group and the down-regulated miRNAs have high expression in low score group. And the patients with high score tended to have poor overall survival. Kaplan-Meier curves for the two groups were shown in Figure 3B. ER-positive patients in high score group suffered worse overall survival than those in low score group (P = 0.022).

In the testing set, similar expression distribution of the miRNAs was found when the cutoff value for each miRNA, the same regression coefficient and cutoff value of risk score derived from the cases in the training phase was applied. And high score group is also prone to exhibiting a worse prognosis (Figure 3C). As shown in Figure 3D, prognosis of cases with high score was significantly worse than those with low score (P = 0.018).

## Discussion

Breast cancer is the most common malignancy and the second leading cause of cancer death among women worldwide [26]. Due to the distinct clinical, pathological and molecular features of the disease, the treatment, response to therapy and corresponding clinical outcome varies greatly [3]. With the help of molecular profiling and the identification of intrinsic subtypes by specific genes, breast cancer patients could benefit from appropriate treatment [27]. ER status is one of the strong factors in predicting patients’ response to endocrine therapy and its determination has become a standard practice in the management of breast cancer [28]. The level of ER was positively correlated with the sensitivity of the endocrine therapy and could predict tamoxifen resistance in breast cancer [29]. However, ER-positive patients are less chemosensitive than ER-negative cases [30] so that adjuvant chemotherapy might not be beneficial to some ER-positive breast tumors [11]. And ER-positive patients also have distinct behaviors and outcome due to different molecular features. Thus, a biomarker which could accurately predict clinical outcome in ER-positive patients with breast cancer is needed urgently.

In the present study, we used miRNA expression microarray data from TCGA and divided the data into two cohorts based on the cases from two sequencing platforms. Following the strategy of using the larger cohort as training set, and the smaller one as the validation set [31], we identified 14 miRNAs which were significantly associated with the status of ER both in training and validation set. The optimal cutoff value for each miRNA to discriminate different status of ER was determined by ROC curve. The risk score calculated from expression of each miRNA weighted by regression coefficient B fitted with status of ER might reflect the tendency from positive to negative status of ER. High score might be more likely related to negative status while low score to positive status. To assess the prognostic value of the miRNA signature, the ER-positive cases were divided into high and low score groups according to the risk score. Twelve miRNAs upregulated in ER-negative breast cancer patients exhibited high expression in high score group and two declined miRNAs showed high expression level in low score group. And the ER-positive patients in high score group suffered poorer survival compared with low score group both in the training stage and validation set.

Lowery et al. [32] found that a 6-miRNA signature could predict status of ER, of which only miR-135b was consistently included in the 14-miRNA signature in our study. To some extent, difference of target population and/or the entry criteria might be responsible for the phenomenon. However, it was also reported that elevated miR-18a [33], miR-505 [34], miR-9 and reduced miR-375 [35] were correlated to oestrogen receptor negativity. The results were consistent with our findings. In addition, re-expression of miR-375 could reverse tamoxifen resistance and epithelial-mesenchymal transition-like properties in the established tamoxifen-resistant breast cancer cells [36]. Moreover, high expression of miR-187 in breast cancer could lead to a more aggressive, invasive phenotype and may act as an independent predictor of outcome [37]. Prognostic value of miR-210 has been explored in many cancer types. Breast cancer patients with elevated miR-210 might have a poor outcome [38,39]. Huang et al. [40] found that miR-224 might act as an oncogene by directly suppressing the RKIP tumor suppressor resulting in promoting metastasis of breast cancer. A higher expression of miR-9 is associated with lymph node metastasis [41] and could act as a predictor for local recurrence of breast cancer [35]. However, the other 5 miRNAs were not explored so widely in breast cancer and further researches are required to investigate their complex molecular mechanisms.

The specificity of biomarkers based on a single miRNA is generally poor [25]. Thus, we developed a risk score of combination the 14 miRNAs associated with ER status and multiplied their corresponding weight to survival and found that the score could predict overall survival in ER-positive patients. Better insights into the mechanism of the 14-miRNA signature in breast cancer might contribute to an understanding of the genetic aberrations that are involved in tumor genesis, progression and response to treatment.

In conclusion, the ER associated miRNA signature identified in our study might support a potential predictor to indicate clinical outcome for ER-positive patients and serve as potential molecular targets for new therapeutic strategies, subsequently leading to improved outcomes.

## Additional file

Additional file 1: Figure S1. ROC curve analyses of 14 miRNAs for patients with different status of ER.
