# Supplementary figures and images for: An ER-associated miRNA signature predicts prognosis in ER-positive breast cancer
Source: J Exp Clin Cancer Res. 2014 Nov 6;33(1):94. doi: 10.1186/s13046-014-0094-5 (PMC4232612; doi:10.1186/s13046-014-0094-5)

Figure S1 ROC curve analyses of 14 miRNAs for patients with different status of ER.


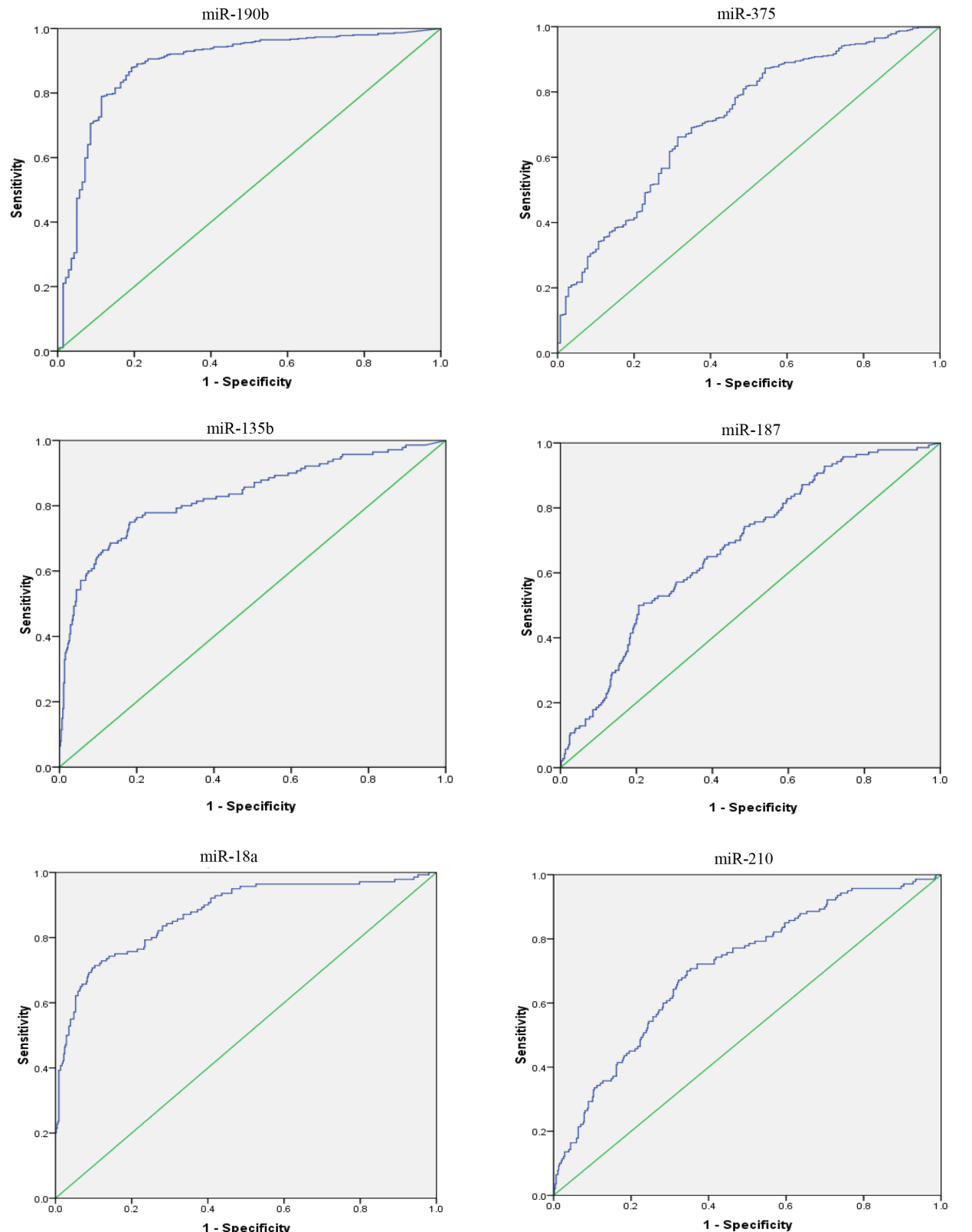


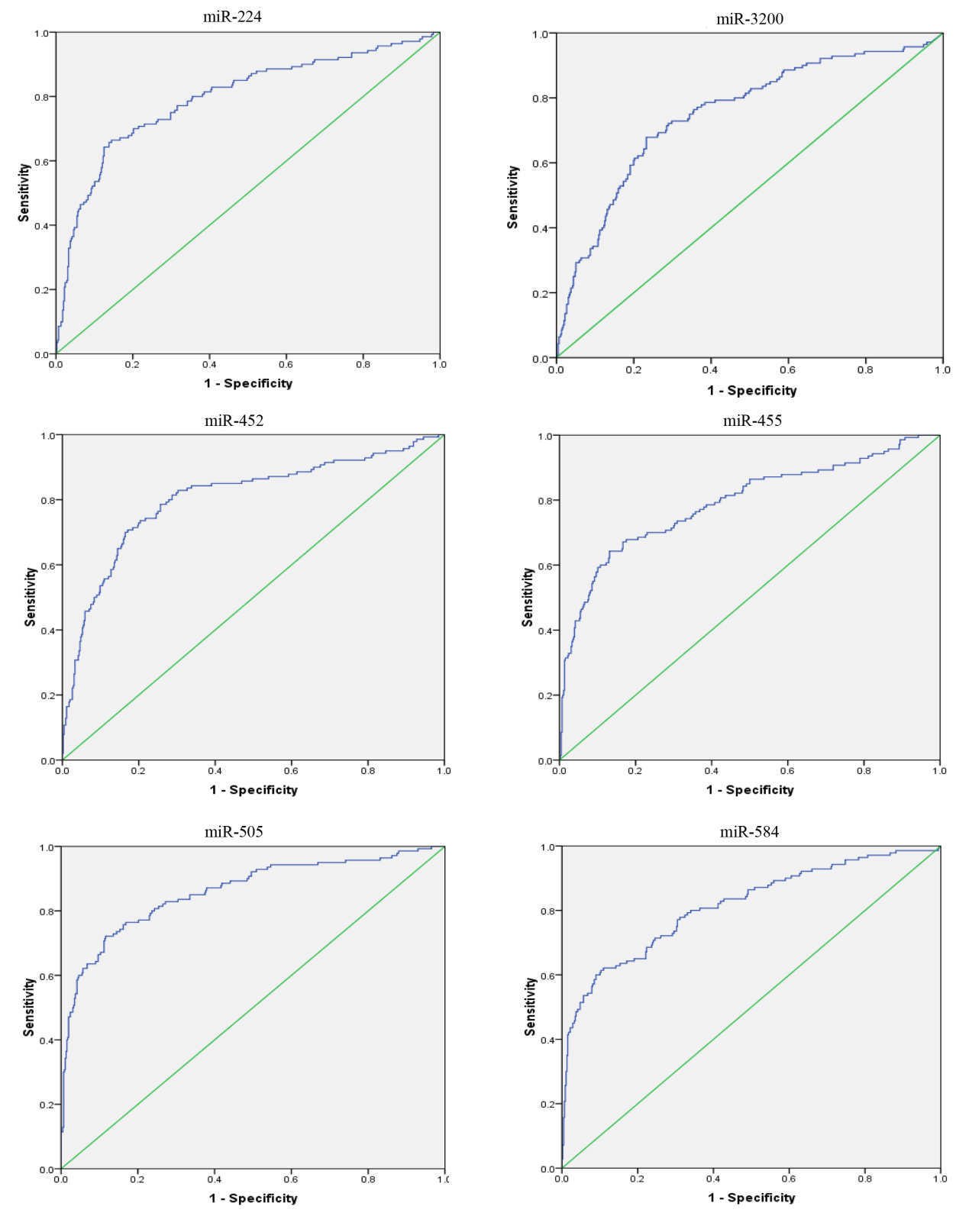


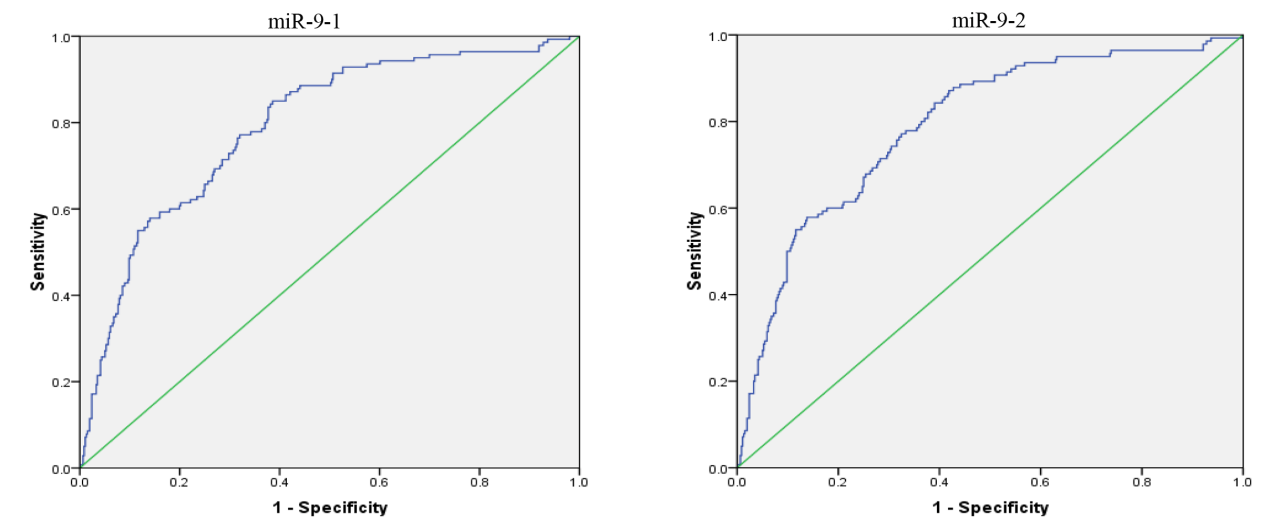

Supplement: Additional file 1: Figure S1. — ROC curve analyses of 14 miRNAs for patients with different status of ER. [file 13046_2014_94_MOESM1_ESM.docx]
